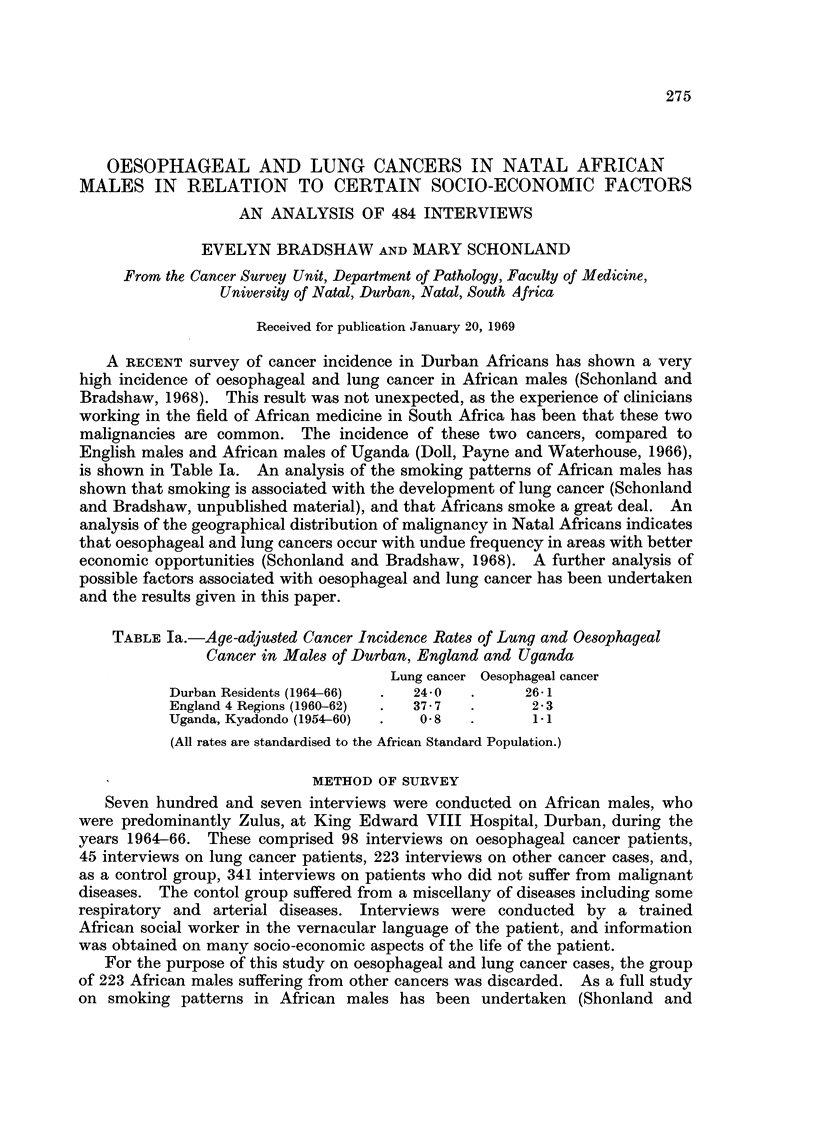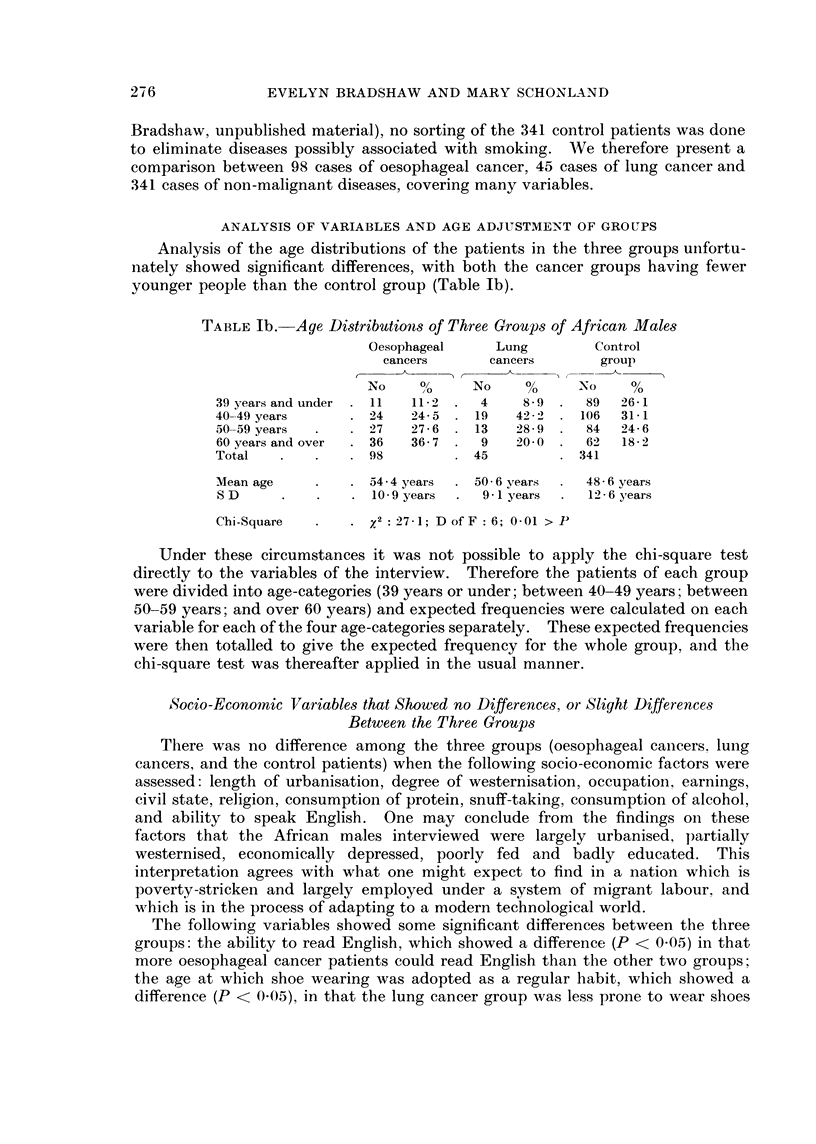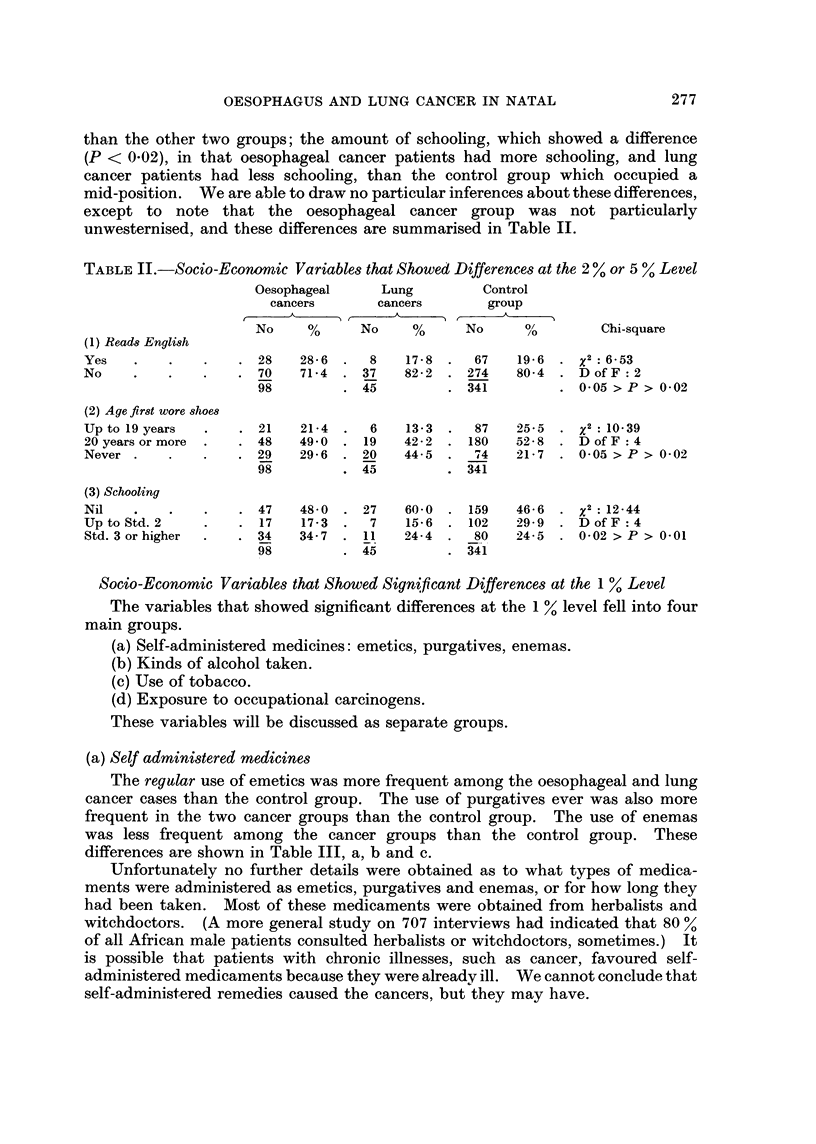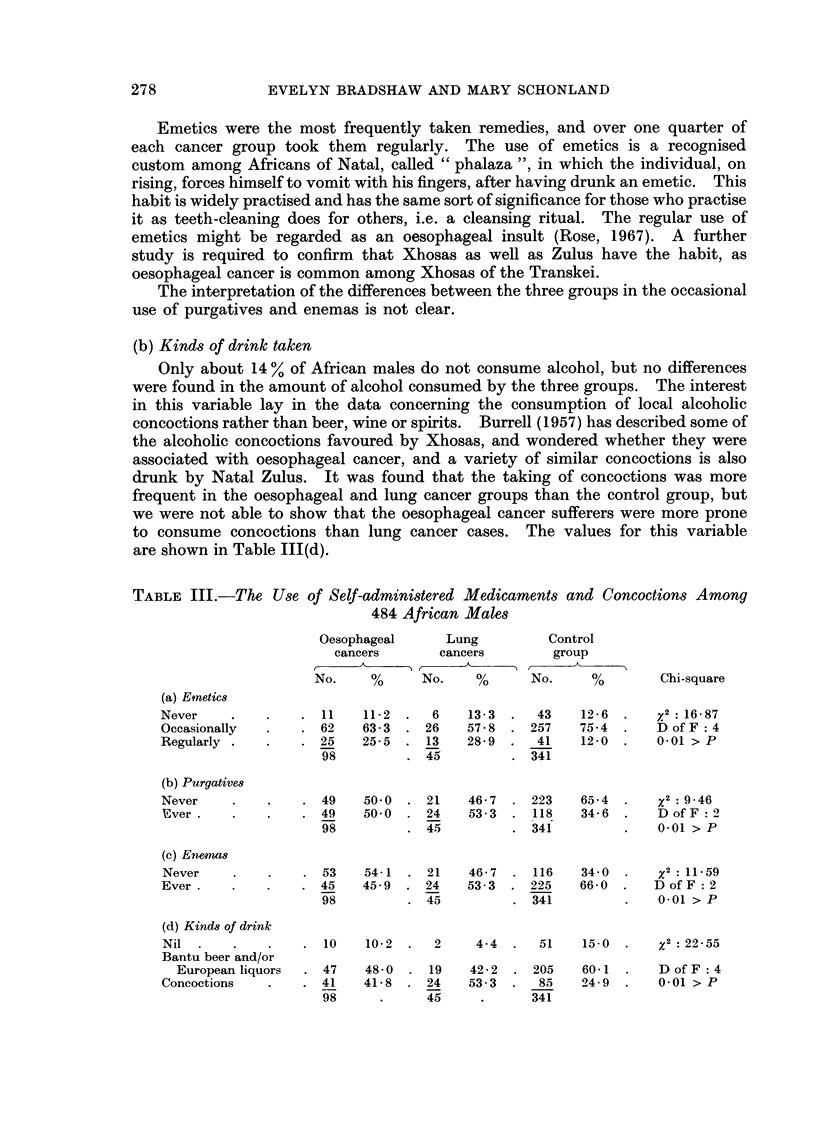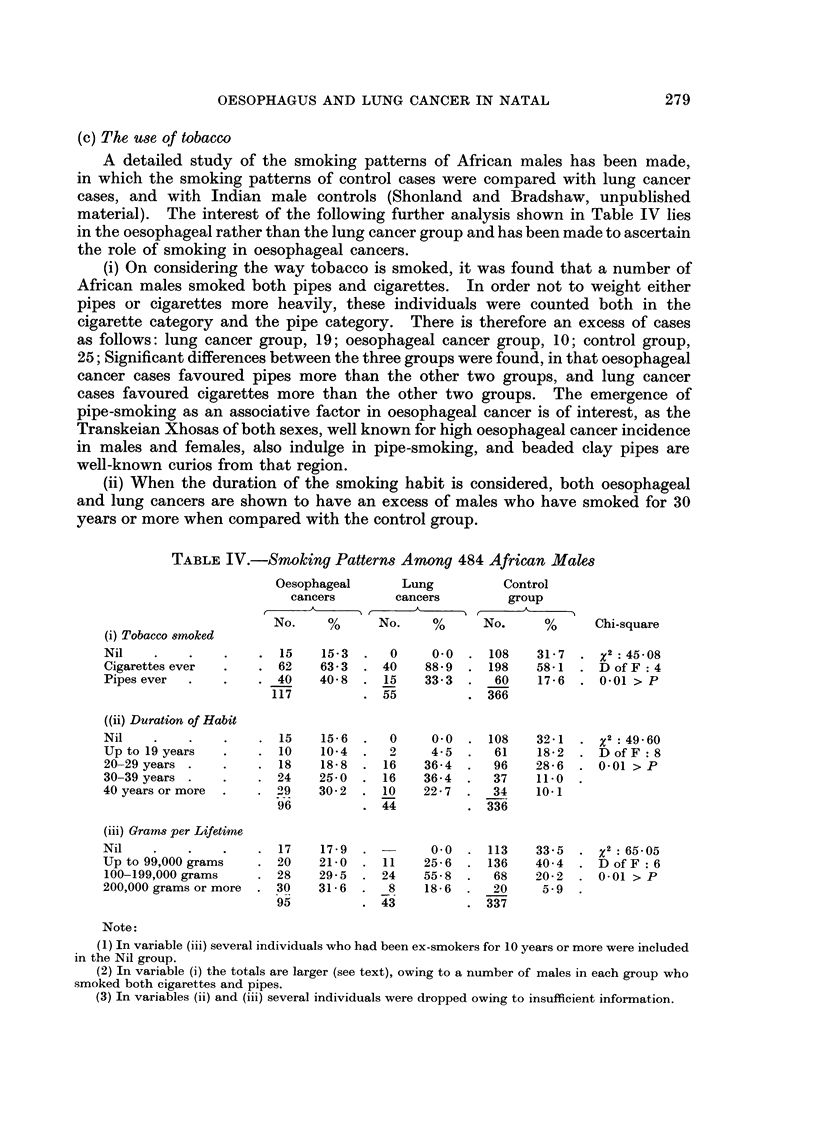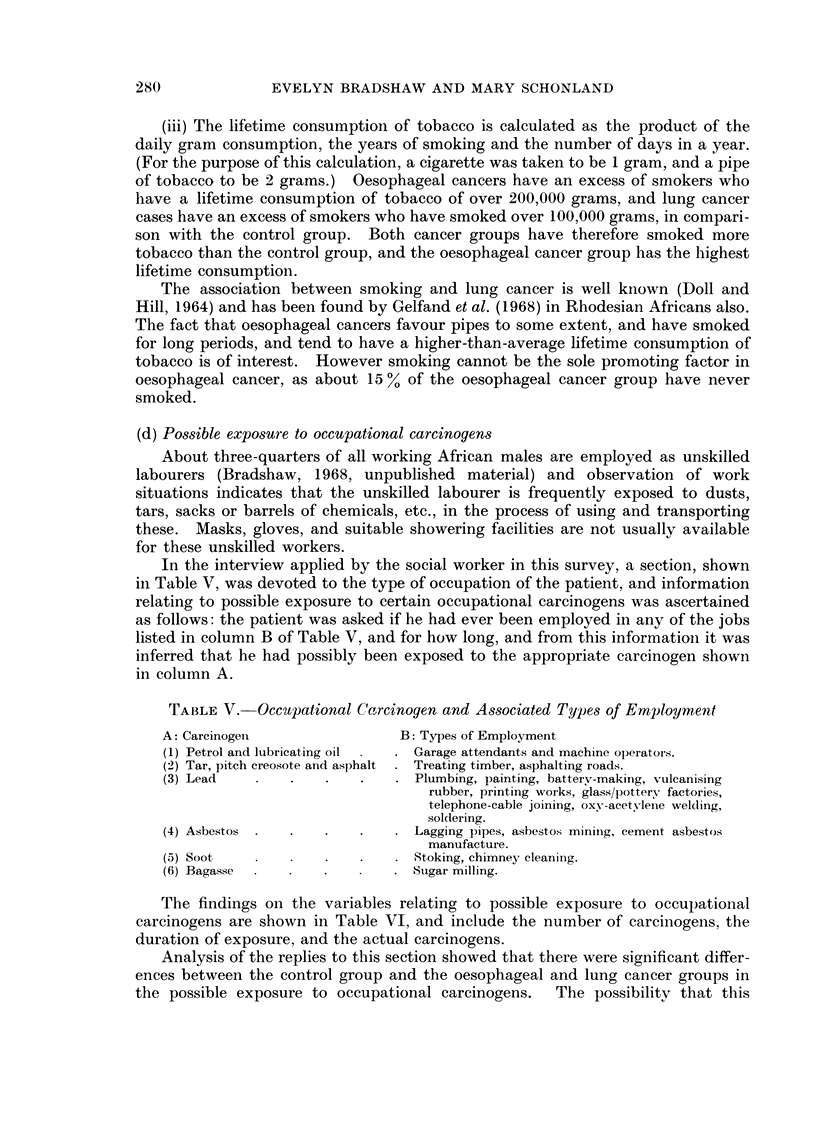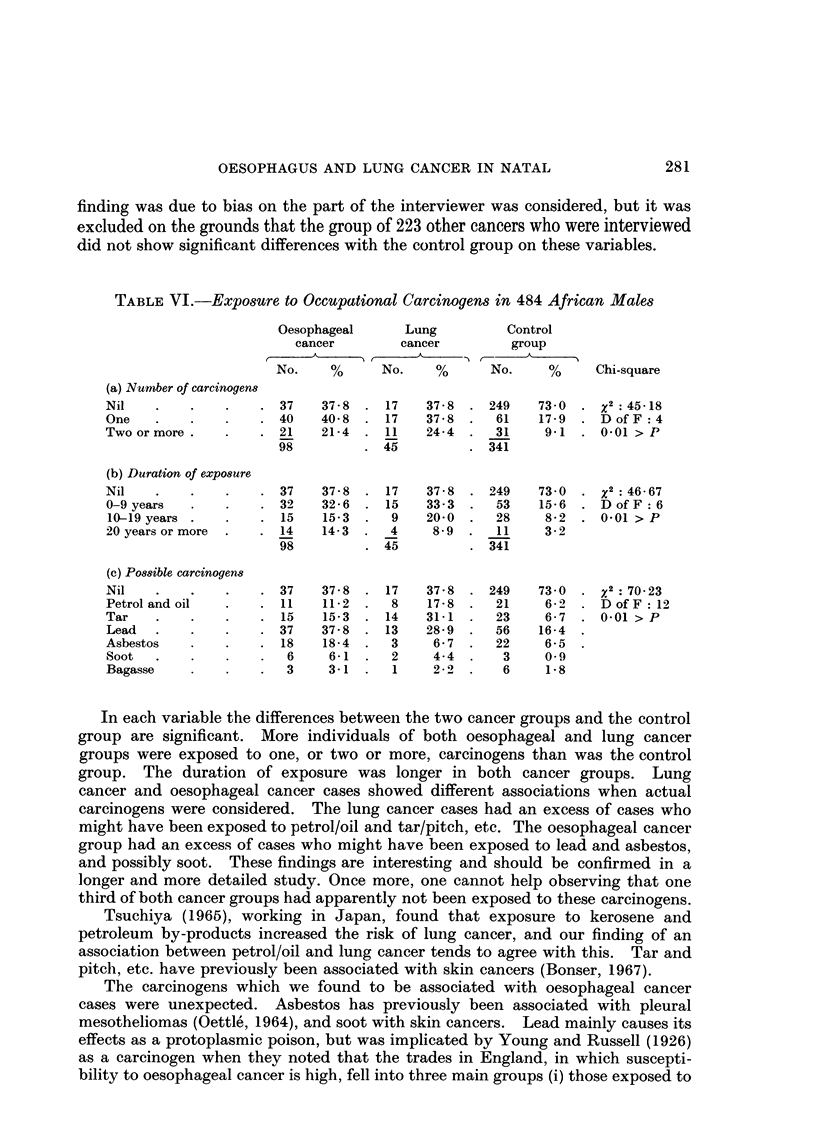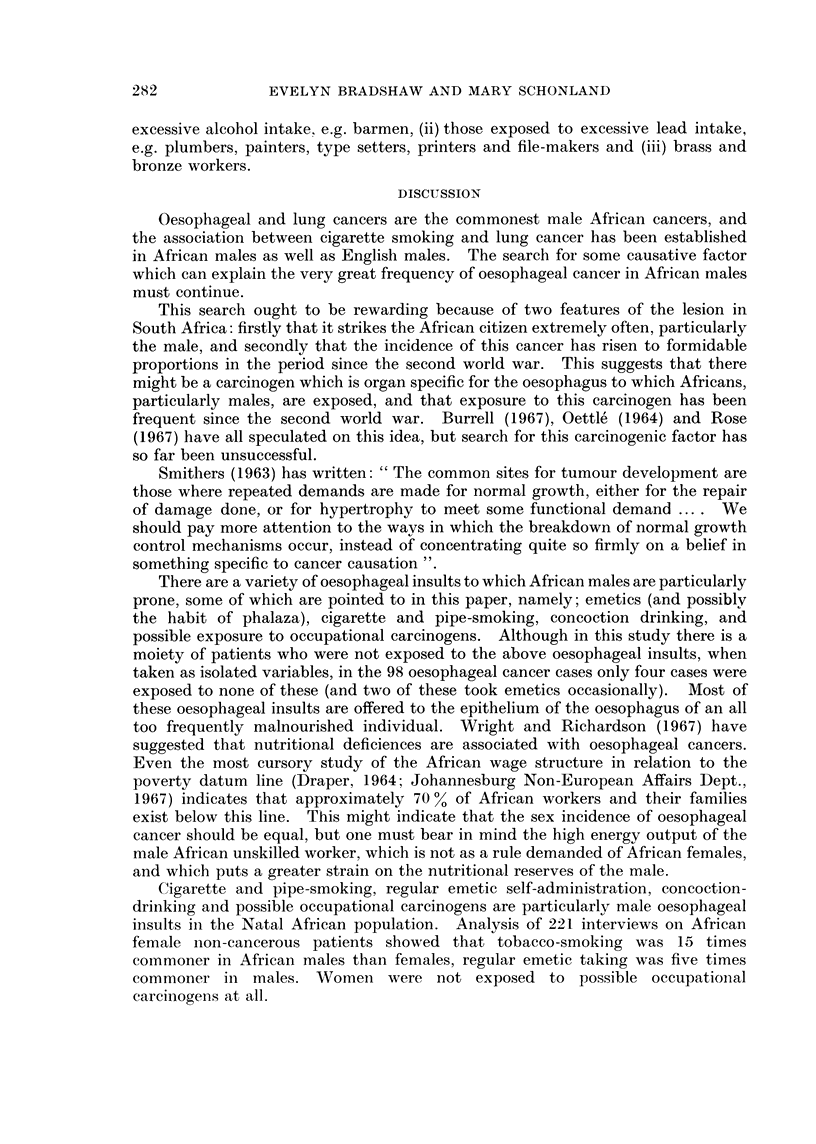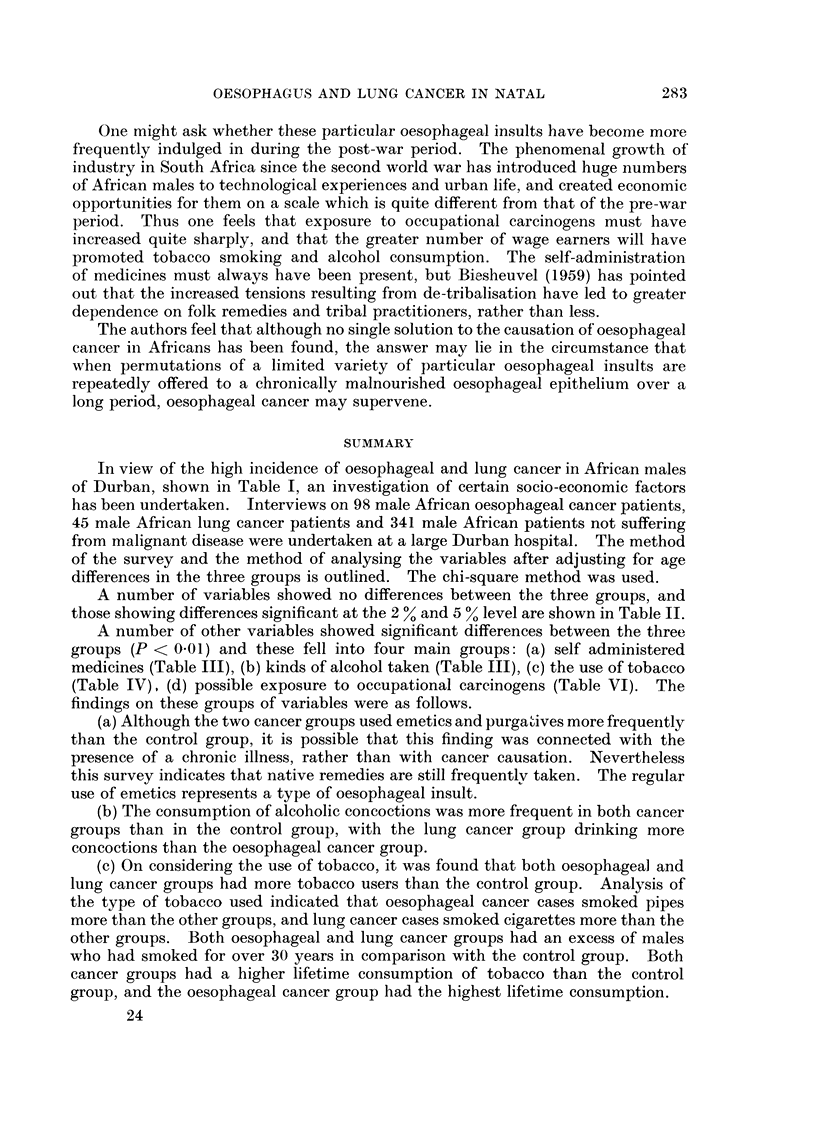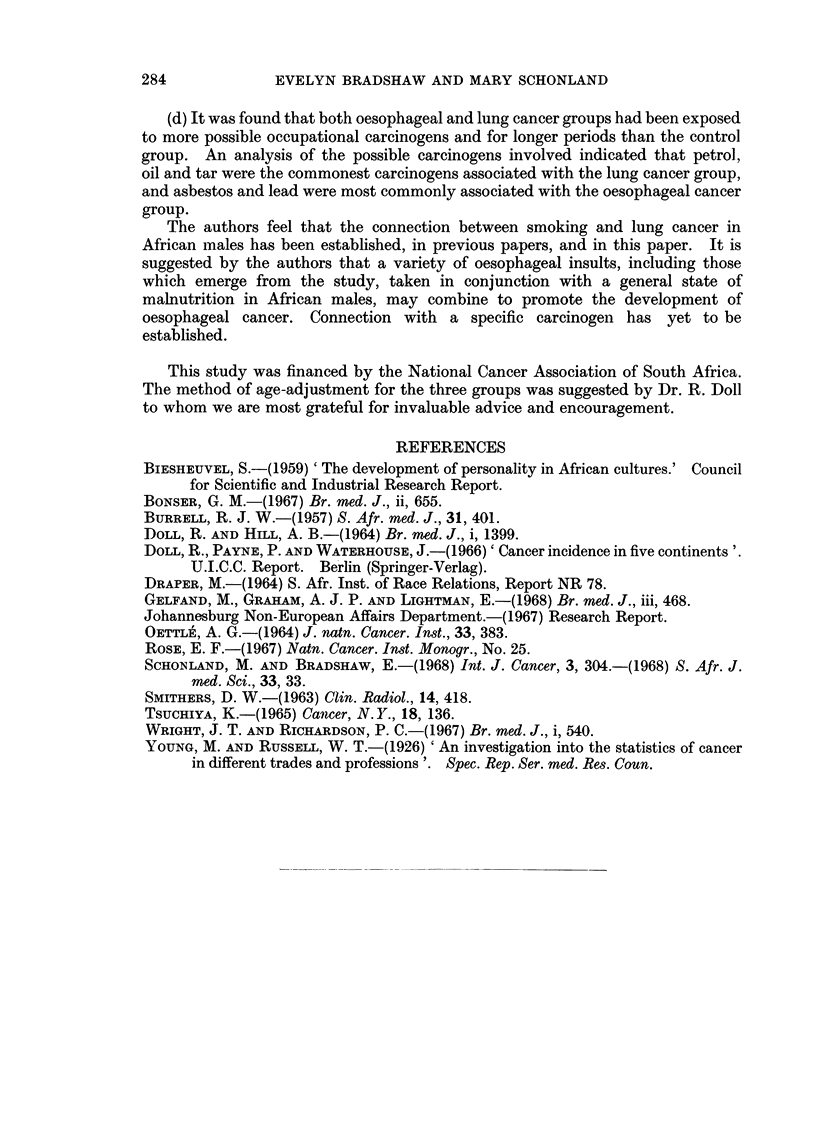# Oesophageal and lung cancers in Natal African males in relation to certain socio-economic factors. An analysis of 484 interviews.

**DOI:** 10.1038/bjc.1969.37

**Published:** 1969-06

**Authors:** E. Bradshaw, M. Schonland


### OESOPHAGEAL AND LUNG CANCERS IN NATAL AFRICAN MALES IN RELATION TO CERTAIN SOCIO-ECONOMIC FACTORS

### AN ANALYSIS OF 484 INTERVIEWS

EVELYN BRADSHAW AND MARY SCHONLAND

*From the Cancer Survey Unit, Department of Pathology, Faculty of Medicine, University of Natal, Durban, Natal, South Africa*

Received for publication January 20, 1969

A RECENT survey of cancer incidence in Durban Africans has shown a very high incidence of oesophageal and lung cancer in African males (Schonland and Bradshaw, 1968). This result was not unexpected, as the experience of clinicians working in the field of African medicine in South Africa has been that these two malignancies are common. The incidence of these two cancers, compared to English males and African males of Uganda (Doll, Payne and Waterhouse, 1966), is shown in Table Ia. An analysis of the smoking patterns of African males has shown that smoking is associated with the development of lung cancer (Schonland and Bradshaw, unpublished material), and that Africans smoke a great deal. An analysis of the geographical distribution of malignancy in Natal Africans indicates that oesophageal and lung cancers occur with undue frequency in areas with better economic opportunities (Schonland and Bradshaw, 1968). A further analysis of possible factors associated with oesophageal and lung cancer has been undertaken and the results given in this paper.

TABLE Ia.—*Age-adjusted Cancer Incidence Rates of Lung and Oesophageal Cancer in Males of Durban, England and Uganda*

| | Lung cancer | Oesophageal cancer |
|---|---|---|
| Durban Residents (1964–66) | 24·0 | 26·1 |
| England 4 Regions (1960–62) | 37·7 | 2·3 |
| Uganda, Kyadondo (1954–60) | 0·8 | 1·1 |

(All rates are standardised to the African Standard Population.)

### METHOD OF SURVEY

Seven hundred and seven interviews were conducted on African males, who were predominantly Zulus, at King Edward VIII Hospital, Durban, during the years 1964–66. These comprised 98 interviews on oesophageal cancer patients, 45 interviews on lung cancer patients, 223 interviews on other cancer cases, and, as a control group, 341 interviews on patients who did not suffer from malignant diseases. The contol group suffered from a miscellany of diseases including some respiratory and arterial diseases. Interviews were conducted by a trained African social worker in the vernacular language of the patient, and information was obtained on many socio-economic aspects of the life of the patient.

For the purpose of this study on oesophageal and lung cancer cases, the group of 223 African males suffering from other cancers was discarded. As a full study on smoking patterns in African males has been undertaken (Shonland and

Bradshaw, unpublished material), no sorting of the 341 control patients was done to eliminate diseases possibly associated with smoking. We therefore present a comparison between 98 cases of oesophageal cancer, 45 cases of lung cancer and 341 cases of non-malignant diseases, covering many variables.

### ANALYSIS OF VARIABLES AND AGE ADJUSTMENT OF GROUPS

Analysis of the age distributions of the patients in the three groups unfortunately showed significant differences, with both the cancer groups having fewer younger people than the control group (Table Ib).

TABLE Ib.—*Age Distributions of Three Groups of African Males*

| | Oesophageal cancers | | Lung cancers | | Control group | |
|---|---|---|---|---|---|---|
| | No | % | No | % | No | % |
| 39 years and under | 11 | 11·2 | 4 | 8·9 | 89 | 26·1 |
| 40–49 years | 24 | 24·5 | 19 | 42·2 | 106 | 31·1 |
| 50–59 years | 27 | 27·6 | 13 | 28·9 | 84 | 24·6 |
| 60 years and over | 36 | 36·7 | 9 | 20·0 | 62 | 18·2 |
| Total | 98 | | 45 | | 341 | |
| Mean age | 54·4 years | | 50·6 years | | 48·6 years | |
| S D | 10·9 years | | 9·1 years | | 12·6 years | |
| Chi-Square | $\chi^2$ : 27·1; D of F : 6; 0·01 > $P$ | | | | | |

Under these circumstances it was not possible to apply the chi-square test directly to the variables of the interview. Therefore the patients of each group were divided into age-categories (39 years or under; between 40–49 years; between 50–59 years; and over 60 years) and expected frequencies were calculated on each variable for each of the four age-categories separately. These expected frequencies were then totalled to give the expected frequency for the whole group, and the chi-square test was thereafter applied in the usual manner.

#### *Socio-Economic Variables that Showed no Differences, or Slight Differences Between the Three Groups*

There was no difference among the three groups (oesophageal cancers, lung cancers, and the control patients) when the following socio-economic factors were assessed: length of urbanisation, degree of westernisation, occupation, earnings, civil state, religion, consumption of protein, snuff-taking, consumption of alcohol, and ability to speak English. One may conclude from the findings on these factors that the African males interviewed were largely urbanised, partially westernised, economically depressed, poorly fed and badly educated. This interpretation agrees with what one might expect to find in a nation which is poverty-stricken and largely employed under a system of migrant labour, and which is in the process of adapting to a modern technological world.

The following variables showed some significant differences between the three groups: the ability to read English, which showed a difference ($P < 0{\cdot}05$) in that more oesophageal cancer patients could read English than the other two groups; the age at which shoe wearing was adopted as a regular habit, which showed a difference ($P < 0{\cdot}05$), in that the lung cancer group was less prone to wear shoes than the other two groups; the amount of schooling, which showed a difference ($P < 0{\cdot}02$), in that oesophageal cancer patients had more schooling, and lung cancer patients had less schooling, than the control group which occupied a mid-position. We are able to draw no particular inferences about these differences, except to note that the oesophageal cancer group was not particularly unwesternised, and these differences are summarised in Table II.

TABLE II.—*Socio-Economic Variables that Showed Differences at the 2% or 5% Level*

| | Oesophageal cancers | | Lung cancers | | Control group | | |
|---|---|---|---|---|---|---|---|
| | No | % | No | % | No | % | Chi-square |
| (1) *Reads English* | | | | | | | |
| Yes | 28 | 28·6 | 8 | 17·8 | 67 | 19·6 | $\chi^2$ : 6·53 |
| No | 70 | 71·4 | 37 | 82·2 | 274 | 80·4 | D of F : 2 |
| | 98 | | 45 | | 341 | | $0{\cdot}05 > P > 0{\cdot}02$ |
| (2) *Age first wore shoes* | | | | | | | |
| Up to 19 years | 21 | 21·4 | 6 | 13·3 | 87 | 25·5 | $\chi^2$ : 10·39 |
| 20 years or more | 48 | 49·0 | 19 | 42·2 | 180 | 52·8 | D of F : 4 |
| Never | 29 | 29·6 | 20 | 44·5 | 74 | 21·7 | $0{\cdot}05 > P > 0{\cdot}02$ |
| | 98 | | 45 | | 341 | | |
| (3) *Schooling* | | | | | | | |
| Nil | 47 | 48·0 | 27 | 60·0 | 159 | 46·6 | $\chi^2$ : 12·44 |
| Up to Std. 2 | 17 | 17·3 | 7 | 15·6 | 102 | 29·9 | D of F : 4 |
| Std. 3 or higher | 34 | 34·7 | 11 | 24·4 | 80 | 24·5 | $0{\cdot}02 > P > 0{\cdot}01$ |
| | 98 | | 45 | | 341 | | |

### *Socio-Economic Variables that Showed Significant Differences at the 1% Level*

The variables that showed significant differences at the 1% level fell into four main groups.

(a) Self-administered medicines: emetics, purgatives, enemas.
(b) Kinds of alcohol taken.
(c) Use of tobacco.
(d) Exposure to occupational carcinogens.

These variables will be discussed as separate groups.

### (a) *Self administered medicines*

The *regular* use of emetics was more frequent among the oesophageal and lung cancer cases than the control group. The use of purgatives ever was also more frequent in the two cancer groups than the control group. The use of enemas was less frequent among the cancer groups than the control group. These differences are shown in Table III, a, b and c.

Unfortunately no further details were obtained as to what types of medicaments were administered as emetics, purgatives and enemas, or for how long they had been taken. Most of these medicaments were obtained from herbalists and witchdoctors. (A more general study on 707 interviews had indicated that 80% of all African male patients consulted herbalists or witchdoctors, sometimes.) It is possible that patients with chronic illnesses, such as cancer, favoured self-administered medicaments because they were already ill. We cannot conclude that self-administered remedies caused the cancers, but they may have.

Emetics were the most frequently taken remedies, and over one quarter of each cancer group took them regularly. The use of emetics is a recognised custom among Africans of Natal, called " phalaza ", in which the individual, on rising, forces himself to vomit with his fingers, after having drunk an emetic. This habit is widely practised and has the same sort of significance for those who practise it as teeth-cleaning does for others, i.e. a cleansing ritual. The regular use of emetics might be regarded as an oesophageal insult (Rose, 1967). A further study is required to confirm that Xhosas as well as Zulus have the habit, as oesophageal cancer is common among Xhosas of the Transkei.

The interpretation of the differences between the three groups in the occasional use of purgatives and enemas is not clear.

(b) *Kinds of drink taken*

Only about 14% of African males do not consume alcohol, but no differences were found in the amount of alcohol consumed by the three groups. The interest in this variable lay in the data concerning the consumption of local alcoholic concoctions rather than beer, wine or spirits. Burrell (1957) has described some of the alcoholic concoctions favoured by Xhosas, and wondered whether they were associated with oesophageal cancer, and a variety of similar concoctions is also drunk by Natal Zulus. It was found that the taking of concoctions was more frequent in the oesophageal and lung cancer groups than the control group, but we were not able to show that the oesophageal cancer sufferers were more prone to consume concoctions than lung cancer cases. The values for this variable are shown in Table III(d).

TABLE III.—*The Use of Self-administered Medicaments and Concoctions Among 484 African Males*

| | Oesophageal cancers | | Lung cancers | | Control group | | |
|---|---|---|---|---|---|---|---|
| | No. | % | No. | % | No. | % | Chi-square |
| (a) *Emetics* | | | | | | | |
| Never | 11 | 11·2 | 6 | 13·3 | 43 | 12·6 | $\chi^2$ : 16·87 |
| Occasionally | 62 | 63·3 | 26 | 57·8 | 257 | 75·4 | D of F : 4 |
| Regularly | 25 | 25·5 | 13 | 28·9 | 41 | 12·0 | 0·01 > $P$ |
| | 98 | | 45 | | 341 | | |
| (b) *Purgatives* | | | | | | | |
| Never | 49 | 50·0 | 21 | 46·7 | 223 | 65·4 | $\chi^2$ : 9·46 |
| Ever | 49 | 50·0 | 24 | 53·3 | 118 | 34·6 | D of F : 2 |
| | 98 | | 45 | | 341 | | 0·01 > $P$ |
| (c) *Enemas* | | | | | | | |
| Never | 53 | 54·1 | 21 | 46·7 | 116 | 34·0 | $\chi^2$ : 11·59 |
| Ever | 45 | 45·9 | 24 | 53·3 | 225 | 66·0 | D of F : 2 |
| | 98 | | 45 | | 341 | | 0·01 > $P$ |
| (d) *Kinds of drink* | | | | | | | |
| Nil | 10 | 10·2 | 2 | 4·4 | 51 | 15·0 | $\chi^2$ : 22·55 |
| Bantu beer and/or European liquors | 47 | 48·0 | 19 | 42·2 | 205 | 60·1 | D of F : 4 |
| Concoctions | 41 | 41·8 | 24 | 53·3 | 85 | 24·9 | 0·01 > $P$ |
| | 98 | | 45 | | 341 | | |

(c) *The use of tobacco*

A detailed study of the smoking patterns of African males has been made, in which the smoking patterns of control cases were compared with lung cancer cases, and with Indian male controls (Shonland and Bradshaw, unpublished material). The interest of the following further analysis shown in Table IV lies in the oesophageal rather than the lung cancer group and has been made to ascertain the role of smoking in oesophageal cancers.

(i) On considering the way tobacco is smoked, it was found that a number of African males smoked both pipes and cigarettes. In order not to weight either pipes or cigarettes more heavily, these individuals were counted both in the cigarette category and the pipe category. There is therefore an excess of cases as follows: lung cancer group, 19; oesophageal cancer group, 10; control group, 25; Significant differences between the three groups were found, in that oesophageal cancer cases favoured pipes more than the other two groups, and lung cancer cases favoured cigarettes more than the other two groups. The emergence of pipe-smoking as an associative factor in oesophageal cancer is of interest, as the Transkeian Xhosas of both sexes, well known for high oesophageal cancer incidence in males and females, also indulge in pipe-smoking, and beaded clay pipes are well-known curios from that region.

(ii) When the duration of the smoking habit is considered, both oesophageal and lung cancers are shown to have an excess of males who have smoked for 30 years or more when compared with the control group.

TABLE IV.—*Smoking Patterns Among* 484 *African Males*

| | Oesophageal cancers | | Lung cancers | | Control group | | |
|---|---|---|---|---|---|---|---|
| | No. | % | No. | % | No. | % | Chi-square |
| (i) *Tobacco smoked* | | | | | | | |
| Nil | 15 | 15·3 | 0 | 0·0 | 108 | 31·7 | $\chi^2$ : 45·08 |
| Cigarettes ever | 62 | 63·3 | 40 | 88·9 | 198 | 58·1 | D of F : 4 |
| Pipes ever | 40 | 40·8 | 15 | 33·3 | 60 | 17·6 | $0 \cdot 01 > P$ |
| | 117 | | 55 | | 366 | | |
| ((ii) *Duration of Habit* | | | | | | | |
| Nil | 15 | 15·6 | 0 | 0·0 | 108 | 32·1 | $\chi^2$ : 49·60 |
| Up to 19 years | 10 | 10·4 | 2 | 4·5 | 61 | 18·2 | D of F : 8 |
| 20–29 years | 18 | 18·8 | 16 | 36·4 | 96 | 28·6 | $0 \cdot 01 > P$ |
| 30–39 years | 24 | 25·0 | 16 | 36·4 | 37 | 11·0 | |
| 40 years or more | 29 | 30·2 | 10 | 22·7 | 34 | 10·1 | |
| | 96 | | 44 | | 336 | | |
| (iii) *Grams per Lifetime* | | | | | | | |
| Nil | 17 | 17·9 | — | 0·0 | 113 | 33·5 | $\chi^2$ : 65·05 |
| Up to 99,000 grams | 20 | 21·0 | 11 | 25·6 | 136 | 40·4 | D of F : 6 |
| 100–199,000 grams | 28 | 29·5 | 24 | 55·8 | 68 | 20·2 | $0 \cdot 01 > P$ |
| 200,000 grams or more | 30 | 31·6 | 8 | 18·6 | 20 | 5·9 | |
| | 95 | | 43 | | 337 | | |

Note:

(1) In variable (iii) several individuals who had been ex-smokers for 10 years or more were included in the Nil group.

(2) In variable (i) the totals are larger (see text), owing to a number of males in each group who smoked both cigarettes and pipes.

(3) In variables (ii) and (iii) several individuals were dropped owing to insufficient information.

(iii) The lifetime consumption of tobacco is calculated as the product of the daily gram consumption, the years of smoking and the number of days in a year. (For the purpose of this calculation, a cigarette was taken to be 1 gram, and a pipe of tobacco to be 2 grams.) Oesophageal cancers have an excess of smokers who have a lifetime consumption of tobacco of over 200,000 grams, and lung cancer cases have an excess of smokers who have smoked over 100,000 grams, in comparison with the control group. Both cancer groups have therefore smoked more tobacco than the control group, and the oesophageal cancer group has the highest lifetime consumption.

The association between smoking and lung cancer is well known (Doll and Hill, 1964) and has been found by Gelfand *et al.* (1968) in Rhodesian Africans also. The fact that oesophageal cancers favour pipes to some extent, and have smoked for long periods, and tend to have a higher-than-average lifetime consumption of tobacco is of interest. However smoking cannot be the sole promoting factor in oesophageal cancer, as about 15% of the oesophageal cancer group have never smoked.

(d) *Possible exposure to occupational carcinogens*

About three-quarters of all working African males are employed as unskilled labourers (Bradshaw, 1968, unpublished material) and observation of work situations indicates that the unskilled labourer is frequently exposed to dusts, tars, sacks or barrels of chemicals, etc., in the process of using and transporting these. Masks, gloves, and suitable showering facilities are not usually available for these unskilled workers.

In the interview applied by the social worker in this survey, a section, shown in Table V, was devoted to the type of occupation of the patient, and information relating to possible exposure to certain occupational carcinogens was ascertained as follows: the patient was asked if he had ever been employed in any of the jobs listed in column B of Table V, and for how long, and from this information it was inferred that he had possibly been exposed to the appropriate carcinogen shown in column A.

TABLE V.—*Occupational Carcinogen and Associated Types of Employment*

| A: Carcinogen | B: Types of Employment |
|---|---|
| (1) Petrol and lubricating oil | Garage attendants and machine operators. |
| (2) Tar, pitch creosote and asphalt | Treating timber, asphalting roads. |
| (3) Lead | Plumbing, painting, battery-making, vulcanising rubber, printing works, glass/pottery factories, telephone-cable joining, oxy-acetylene welding, soldering. |
| (4) Asbestos | Lagging pipes, asbestos mining, cement asbestos manufacture. |
| (5) Soot | Stoking, chimney cleaning. |
| (6) Bagasse | Sugar milling. |

The findings on the variables relating to possible exposure to occupational carcinogens are shown in Table VI, and include the number of carcinogens, the duration of exposure, and the actual carcinogens.

Analysis of the replies to this section showed that there were significant differences between the control group and the oesophageal and lung cancer groups in the possible exposure to occupational carcinogens. The possibility that this finding was due to bias on the part of the interviewer was considered, but it was excluded on the grounds that the group of 223 other cancers who were interviewed did not show significant differences with the control group on these variables.

TABLE VI.—*Exposure to Occupational Carcinogens in* 484 *African Males*

| | Oesophageal cancer | | Lung cancer | | Control group | | |
|---|---|---|---|---|---|---|---|
| | No. | % | No. | % | No. | % | Chi-square |
| (a) *Number of carcinogens* | | | | | | | |
| Nil | 37 | 37·8 | 17 | 37·8 | 249 | 73·0 | $\chi^2$ : 45·18 |
| One | 40 | 40·8 | 17 | 37·8 | 61 | 17·9 | D of F : 4 |
| Two or more | 21 | 21·4 | 11 | 24·4 | 31 | 9·1 | $0{\cdot}01 > P$ |
| | 98 | | 45 | | 341 | | |
| (b) *Duration of exposure* | | | | | | | |
| Nil | 37 | 37·8 | 17 | 37·8 | 249 | 73·0 | $\chi^2$ : 46·67 |
| 0–9 years | 32 | 32·6 | 15 | 33·3 | 53 | 15·6 | D of F : 6 |
| 10–19 years | 15 | 15·3 | 9 | 20·0 | 28 | 8·2 | $0{\cdot}01 > P$ |
| 20 years or more | 14 | 14·3 | 4 | 8·9 | 11 | 3·2 | |
| | 98 | | 45 | | 341 | | |
| (c) *Possible carcinogens* | | | | | | | |
| Nil | 37 | 37·8 | 17 | 37·8 | 249 | 73·0 | $\chi^2$ : 70·23 |
| Petrol and oil | 11 | 11·2 | 8 | 17·8 | 21 | 6·2 | D of F : 12 |
| Tar | 15 | 15·3 | 14 | 31·1 | 23 | 6·7 | $0{\cdot}01 > P$ |
| Lead | 37 | 37·8 | 13 | 28·9 | 56 | 16·4 | |
| Asbestos | 18 | 18·4 | 3 | 6·7 | 22 | 6·5 | |
| Soot | 6 | 6·1 | 2 | 4·4 | 3 | 0·9 | |
| Bagasse | 3 | 3·1 | 1 | 2·2 | 6 | 1·8 | |

In each variable the differences between the two cancer groups and the control group are significant. More individuals of both oesophageal and lung cancer groups were exposed to one, or two or more, carcinogens than was the control group. The duration of exposure was longer in both cancer groups. Lung cancer and oesophageal cancer cases showed different associations when actual carcinogens were considered. The lung cancer cases had an excess of cases who might have been exposed to petrol/oil and tar/pitch, etc. The oesophageal cancer group had an excess of cases who might have been exposed to lead and asbestos, and possibly soot. These findings are interesting and should be confirmed in a longer and more detailed study. Once more, one cannot help observing that one third of both cancer groups had apparently not been exposed to these carcinogens.

Tsuchiya (1965), working in Japan, found that exposure to kerosene and petroleum by-products increased the risk of lung cancer, and our finding of an association between petrol/oil and lung cancer tends to agree with this. Tar and pitch, etc. have previously been associated with skin cancers (Bonser, 1967).

The carcinogens which we found to be associated with oesophageal cancer cases were unexpected. Asbestos has previously been associated with pleural mesotheliomas (Oettlé, 1964), and soot with skin cancers. Lead mainly causes its effects as a protoplasmic poison, but was implicated by Young and Russell (1926) as a carcinogen when they noted that the trades in England, in which susceptibility to oesophageal cancer is high, fell into three main groups (i) those exposed to excessive alcohol intake, e.g. barmen, (ii) those exposed to excessive lead intake, e.g. plumbers, painters, type setters, printers and file-makers and (iii) brass and bronze workers.

### DISCUSSION

Oesophageal and lung cancers are the commonest male African cancers, and the association between cigarette smoking and lung cancer has been established in African males as well as English males. The search for some causative factor which can explain the very great frequency of oesophageal cancer in African males must continue.

This search ought to be rewarding because of two features of the lesion in South Africa: firstly that it strikes the African citizen extremely often, particularly the male, and secondly that the incidence of this cancer has risen to formidable proportions in the period since the second world war. This suggests that there might be a carcinogen which is organ specific for the oesophagus to which Africans, particularly males, are exposed, and that exposure to this carcinogen has been frequent since the second world war. Burrell (1967), Oettlé (1964) and Rose (1967) have all speculated on this idea, but search for this carcinogenic factor has so far been unsuccessful.

Smithers (1963) has written: " The common sites for tumour development are those where repeated demands are made for normal growth, either for the repair of damage done, or for hypertrophy to meet some functional demand .... We should pay more attention to the ways in which the breakdown of normal growth control mechanisms occur, instead of concentrating quite so firmly on a belief in something specific to cancer causation ".

There are a variety of oesophageal insults to which African males are particularly prone, some of which are pointed to in this paper, namely; emetics (and possibly the habit of phalaza), cigarette and pipe-smoking, concoction drinking, and possible exposure to occupational carcinogens. Although in this study there is a moiety of patients who were not exposed to the above oesophageal insults, when taken as isolated variables, in the 98 oesophageal cancer cases only four cases were exposed to none of these (and two of these took emetics occasionally). Most of these oesophageal insults are offered to the epithelium of the oesophagus of an all too frequently malnourished individual. Wright and Richardson (1967) have suggested that nutritional deficiences are associated with oesophageal cancers. Even the most cursory study of the African wage structure in relation to the poverty datum line (Draper, 1964; Johannesburg Non-European Affairs Dept., 1967) indicates that approximately 70 % of African workers and their families exist below this line. This might indicate that the sex incidence of oesophageal cancer should be equal, but one must bear in mind the high energy output of the male African unskilled worker, which is not as a rule demanded of African females, and which puts a greater strain on the nutritional reserves of the male.

Cigarette and pipe-smoking, regular emetic self-administration, concoction-drinking and possible occupational carcinogens are particularly male oesophageal insults in the Natal African population. Analysis of 221 interviews on African female non-cancerous patients showed that tobacco-smoking was 15 times commoner in African males than females, regular emetic taking was five times commoner in males. Women were not exposed to possible occupational carcinogens at all.

One might ask whether these particular oesophageal insults have become more frequently indulged in during the post-war period. The phenomenal growth of industry in South Africa since the second world war has introduced huge numbers of African males to technological experiences and urban life, and created economic opportunities for them on a scale which is quite different from that of the pre-war period. Thus one feels that exposure to occupational carcinogens must have increased quite sharply, and that the greater number of wage earners will have promoted tobacco smoking and alcohol consumption. The self-administration of medicines must always have been present, but Biesheuvel (1959) has pointed out that the increased tensions resulting from de-tribalisation have led to greater dependence on folk remedies and tribal practitioners, rather than less.

The authors feel that although no single solution to the causation of oesophageal cancer in Africans has been found, the answer may lie in the circumstance that when permutations of a limited variety of particular oesophageal insults are repeatedly offered to a chronically malnourished oesophageal epithelium over a long period, oesophageal cancer may supervene.

### SUMMARY

In view of the high incidence of oesophageal and lung cancer in African males of Durban, shown in Table I, an investigation of certain socio-economic factors has been undertaken. Interviews on 98 male African oesophageal cancer patients, 45 male African lung cancer patients and 341 male African patients not suffering from malignant disease were undertaken at a large Durban hospital. The method of the survey and the method of analysing the variables after adjusting for age differences in the three groups is outlined. The chi-square method was used.

A number of variables showed no differences between the three groups, and those showing differences significant at the 2 % and 5 % level are shown in Table II.

A number of other variables showed significant differences between the three groups ($P < 0{\cdot}01$) and these fell into four main groups: (a) self administered medicines (Table III), (b) kinds of alcohol taken (Table III), (c) the use of tobacco (Table IV), (d) possible exposure to occupational carcinogens (Table VI). The findings on these groups of variables were as follows.

(a) Although the two cancer groups used emetics and purgatives more frequently than the control group, it is possible that this finding was connected with the presence of a chronic illness, rather than with cancer causation. Nevertheless this survey indicates that native remedies are still frequently taken. The regular use of emetics represents a type of oesophageal insult.

(b) The consumption of alcoholic concoctions was more frequent in both cancer groups than in the control group, with the lung cancer group drinking more concoctions than the oesophageal cancer group.

(c) On considering the use of tobacco, it was found that both oesophageal and lung cancer groups had more tobacco users than the control group. Analysis of the type of tobacco used indicated that oesophageal cancer cases smoked pipes more than the other groups, and lung cancer cases smoked cigarettes more than the other groups. Both oesophageal and lung cancer groups had an excess of males who had smoked for over 30 years in comparison with the control group. Both cancer groups had a higher lifetime consumption of tobacco than the control group, and the oesophageal cancer group had the highest lifetime consumption.

(d) It was found that both oesophageal and lung cancer groups had been exposed to more possible occupational carcinogens and for longer periods than the control group. An analysis of the possible carcinogens involved indicated that petrol, oil and tar were the commonest carcinogens associated with the lung cancer group, and asbestos and lead were most commonly associated with the oesophageal cancer group.

The authors feel that the connection between smoking and lung cancer in African males has been established, in previous papers, and in this paper. It is suggested by the authors that a variety of oesophageal insults, including those which emerge from the study, taken in conjunction with a general state of malnutrition in African males, may combine to promote the development of oesophageal cancer. Connection with a specific carcinogen has yet to be established.

This study was financed by the National Cancer Association of South Africa. The method of age-adjustment for the three groups was suggested by Dr. R. Doll to whom we are most grateful for invaluable advice and encouragement.

### REFERENCES

BIESHEUVEL, S.—(1959) ' The development of personality in African cultures.' Council for Scientific and Industrial Research Report.
BONSER, G. M.—(1967) *Br. med. J.*, ii, 655.
BURRELL, R. J. W.—(1957) *S. Afr. med. J.*, **31**, 401.
DOLL, R. AND HILL, A. B.—(1964) *Br. med. J.*, i, 1399.
DOLL, R., PAYNE, P. AND WATERHOUSE, J.—(1966) ' Cancer incidence in five continents '. U.I.C.C. Report. Berlin (Springer-Verlag).
DRAPER, M.—(1964) S. Afr. Inst. of Race Relations, Report NR 78.
GELFAND, M., GRAHAM, A. J. P. AND LIGHTMAN, E.—(1968) *Br. med. J.*, iii, 468.
Johannesburg Non-European Affairs Department.—(1967) Research Report.
OETTLÉ, A. G.—(1964) *J. natn. Cancer. Inst.*, **33**, 383.
ROSE, E. F.—(1967) *Natn. Cancer. Inst. Monogr.*, No. 25.
SCHONLAND, M. AND BRADSHAW, E.—(1968) *Int. J. Cancer*, **3**, 304.—(1968) *S. Afr. J. med. Sci.*, **33**, 33.
SMITHERS, D. W.—(1963) *Clin. Radiol.*, **14**, 418.
TSUCHIYA, K.—(1965) *Cancer, N.Y.*, **18**, 136.
WRIGHT, J. T. AND RICHARDSON, P. C.—(1967) *Br. med. J.*, i, 540.
YOUNG, M. AND RUSSELL, W. T.—(1926) ' An investigation into the statistics of cancer in different trades and professions '. *Spec. Rep. Ser. med. Res. Coun.*